# Salinity stress and drought stress differentially affect biomass allocation, leaf orientation and performance of two sesame (*Sesamum indicum* L.) cultivars

**DOI:** 10.1186/s12870-026-09646-9

**Published:** 2026-08-08

**Authors:** Taha Mohamed El-Katony, Nemat Mohamed Hassan, Samia Helmy Abo-Ismael, Shaimaa Nassim Abdelfatah

**Affiliations:** Department of Botany and Microbiology, Faculty of Science, Damietta University, PO box 34517, New Damietta City, Egypt

**Keywords:** Drought, Salinity, Sesame genotypes, Gas exchange, Carbohydrate fractions, Leaf orientation

## Abstract

**Background:**

Drought and salinity stresses are major and interrelated threats to crop productivity in arid lands. Understanding the mechanisms of tolerance to salinity and drought stresses is critical for selection of productive crop cultivars.

**Methods:**

This work investigates the differential impact of salinity and drought stresses on growth and performance of Sohag 1 (Sohg) and Shandaweel 3 (Shnd) cultivars of sesame at the same water potential (0, -100, -250 and − 450 kPa) and nutrient supply in a hydroponic sand culture. Plant response to stress was evaluated in terms of growth and biomass fractionation, leaf orientation and gas exchange as well as the contents and fractionation of leaf photosynthetic pigments and carbohydrates.

**Results:**

The horizontal leaf orientation of Shnd was marginally affected by stress; meanwhile, the vertical leaves of Sohg became more vertical under salinity stress but less vertical under drought stress. Foliage of the two sesame cultivars was more tolerant to salt stress than to drought stress, with better tolerance of Shnd than Sohg. This was evident from the progressive drought-induced inhibition of foliage growth versus a mild retardation or even benefit by low salinity, along with slightly less severe effect of high salinity. Salinity targeted particularly root growth with favored biomass allocation to the foliage. By contrast, the enhancement of root growth and retardation of foliage growth under drought implies favored biomass allocation to root. Salinity reduced leaf pigment content in the two cultivars, particularly Sohg but drought either increased it or was without effect. Stress, post a threshold of -100 kPa, reduced stomatal conductance, the rates of photosynthesis and transpiration and water use efficiency, with stronger impact of salinity than drought. The lowering in leaf carbohydrate content was more aggressive under salinity than under drought, and was associated with increasing proportion of pectin at the expense of starch but variable changes in soluble sugars.

**Conclusion:**

Salinity and drought stresses, at equivalent water potentials, differentially affect sesame growth, gas exchange, leaf orientation, photosynthetic pigments and carbohydrate fractions in a genotype-dependent pattern. Among the two investigated sesame cultivars, Shnd seems more stress-tolerant than Sohg. The present findings, obtained from a hydroponic sand culture under partially protected conditions, needs further evaluation under field conditions employing wider collection of genotypes.

## Introduction

Under natural conditions, plants may experience combined stresses simultaneously, such as the combined stress of high temperature, water deficit and high irradiance in the hot dry summer of arid habitats [1]. Among the different abiotic stresses affecting plants, salinity stress and water stress are particularly serious and mostly interrelated. Generally, saline soils are abundant in semiarid and arid regions where rainfall is too scarce to allow leaching of excessive soluble salts out of the rhizosphere [2]. In turn, usage of poor-quality water in irrigation, a practice common in the arid developing countries, can induce secondary salinization. The soil is categorized as saline when its saturation extract has electrical conductivity > 4 dS m^− 1^ (> 40 mM NaCl), along with exchangeable sodium percentage < 15 [3].

Although the majority of crop plants are salt-sensitive glycophytes, few species are salt-tolerant and can even benefit from mild salinity. Traditionally, two criteria are used to evaluate salt tolerance of crop plants: (1) the threshold salinity and (2) the slope of the salinity-growth response line beyond the threshold. Differences in salt tolerance can occur among different species and cultivars as well as during the different plant developmental stages [4, 5]. Similarly, drought stress represents a major challenge to plant productivity and land use, particularly in the arid regions. Even in the tropical rain forests, land plants can experience different degrees of transient water stress when water absorption lags behind evapotranspiration [6]. Unfortunately, the witnessed global warming will further aggravate the threat of drought to plant productivity. Intrinsically, plants have low water use efficiency, with less than 1% of the absorbed water–versus more than 90% of the absorbed mineral nutrients–participating in biomass gain [1]. Water is translocated within the plant down a gradient of (1) water potential (Ψ_w_) across membranes from the soil to the root and throughout the root, (2) hydrostatic pressure in the xylem vessels and (3) water vapor gradient from the leaf to air.

Under the impact of salinity, plants suffer from several hazards including osmotic stress, ion toxicity/specific ion effect, nutrient imbalance and oxidative stress. The relative participation of either factor depends on the level, duration and mode of exposure to salt stress as well as the plant developmental stage. For example, long-term salinity stress leads mainly to ion toxicity in the older leaves but to water deficit and shortage of carbohydrates in the younger leaves [7]. Salinity and drought stresses cause morphological, physiological, biochemical and molecular alterations in the plant. To cope with stress, the plant has to expend additional energy for ion exclusion or compartmentalization under salinity stress. Likewise, strategies of osmotic adjustment and detoxification of reactive oxygen species under the impact of salinity and drought stresses will slow down plant growth [8]. As a rule, shoot growth is more depressed by salinity and drought than root growth, partially because of favored biomass allocation to roots at the expense of shoots [9].

In addition to the direct toxicity of high levels of Na^+^ and Cl^-^ in the symplasm, salt toxicity can also be attributed to disruption of respiration, photosynthesis and protein synthesis. Salinity can induce shortage of cellular K^+^ concentrations which will impair enzyme activity, protein synthesis, photosynthesis and stomatal movement [10]. The ability of plants to protect enzymes from the adverse effects of Na^+^ or Cl^-^ can be attributed to the synthesis of compatible solutes such as sugars and amino acids (e.g. proline); the latter can also aid in stabilizing the structure of membranes and macromolecules [11]. The role of compatible solutes in plant protection is also evident under drought stress. Upon experiencing drought, plant cells can adjust Ψ_w_ by accumulating inorganic ions and organic acids in the vacuoles; meanwhile, synthesizing compatible organic solutes such as glycine betaine, sorbitol, and proline in the cytoplasm. The salinity-induced reduction in photosynthesis can be attributed to the reductions in leaf area (a capacity factor) and in net CO_2_ fixation per unit leaf area (an efficiency factor) in addition to the decreased demand of sink tissues for assimilates [12]. Further, the effect of salinity on photosynthetic efficiency includes both stomatal and nonstomatal limitations. Stomatal limitations are manifested as lowered intercellular CO_2_ concentration while nonstomatal factors include the decrease in quantum efficiency and inhibited Rubisco activity [13]. Stomatal closure and the lowered stomatal conductance represent a way to prevent excessive dehydration of the leaves as a feedforward response to anticipate water deficit before the progression of severe drought.

Sesame (*Sesamum indicum* L.), a Pedaliaceae member and one of the most valuable oil cash crops in Africa and Asia [14], is a robust crop with wide niche and marked drought tolerance. Sesame has wide genotypic variability regarding yield potentiality, tolerance to abiotic stress and seed contents of oil and protein. The white-seeded Shnd is more vigorous with higher yield potentiality and more germination resistance to NaCl-salinity stress and PEG-water stress than the red-seeded Sohg [15–17]. The high oil content of sesame seeds (up to 63%) is characterized with high proportion of unsaturated fatty acids, particularly omega 3 and γ-tocopherol [18]. Sesame seeds are also rich in human-compatible proteins, vitamins, dietary fibers and dietary minerals, particularly Ca^2+^ [19, 20]. Furthermore, sesame seeds contain bioactive components such as polyphenols, lignans, phytosterols, quinones and triterpenoids [21].

The present work investigates the differential impact of NaCl salinity and water-withholding drought, at the same Ψ_w_, on growth and performance of two Egyptian sesame cultivars: Sohg and Shnd. Salinity stress and drought stress were imposed with the same Ψ_w_ and nutrient supply in a hydroponic sand culture to distinguish the osmotic effect of salinity from the specific ion effect, meanwhile avoiding the complications of inconsistent nutrient supply. Because salinity stress includes both an osmotic effect and an additional specific ion effect, it is expected for salinity stress to be more dangerous to plant life than drought stress. However, there is the possibility that lowering of plant cell Ψ_w_ via uptake of salt ions might, within limits, allow better water absorption under salinity stress relative to the osmotically-equivalent drought stress. Partitioning of biomass, photosynthetic pigments and carbohydrate fractions has been integrated with photosynthetic efficiency to achieve a plausible explanation of the differential plant response to salinity and drought stresses.

Drought stress is routinely imposed either by employing high molecular weight hydrophilic polymers such as poly ethylene glycol (PEG) or by water withholding. The complications of the first route arise from the fact that PEG is an artifact, and its impact on plant performance might involve PEG-specific effects under the premise of its uptake by the root. In turn, water withholding via preventing irrigation in a confined substrate will shorten the duration of investigation and can lead to abrupt plant death. In the present work, manipulation of the hydroponic sand culture with scheduled timely addition of treatment solutions allows providing identical supply of nutrients with identical water potential (Ψ_w_) under drought stress and salinity stress. Nevertheless, the outcomes of this hydroponic study, although arising from a precise procedure, need further evaluation at the field scale because of the discrepancy between root environment in the sand culture and the heterogenous soil substrate.

## Materials and methods

### Plant material

Seeds of cultivars Sohag 1 (Sohg) and Shandaweel 3 (Shnd) of sesame (*Sesamum indicum* L.) were kindly obtained from the Agricultural Research Center (ARC), Egypt. The two experimental sesame cultivars are national cultivars that are produced by Field Crops Research Institute (FCRI), Agricultural Research Center (ARC), Egypt. The two cultivars were produced according to guidelines of the International Union for the Protection of New Varieties of Plants (UPOV). The acquired names “Sohag 1” and “Shandaweel 3” are given by the breeder program of ARC and are related to national districts of Egypt.

### Effect of salinity and drought on sesame growth and performance

Uniform seeds were surface-sterilized in 10% (v/v) Clorox for 20 min, followed by thorough washing with tap water. The seeds were then sown on plastic pots of 15 cm diameter and 20 cm height full of 1.1 kg of water-washed sand, five seeds per pot. To ensure uniform germination, the seeds were initially watered with 0.2 mM CaSO_4_ for 7 days. Thereafter, the seedlings were successively thinned to one per pot and received the nutrient solution in a successive manner. First, seedlings were watered with one-tenth strength nutrient solution for three days, followed by half-strength solution for six days and finally the full-strength solution for 13 days. The full-strength nutrient solution was of the following composition:

- Macronutrients (mM): N (as NO_3_^−^) 16, K 7.5, P 1.5, Ca 5, Mg 1.5 and S 1.5.
- Micronutrients (µM): Fe 50, Mn 5, Cu 0.5, Zn 0.5, B 25, Mo 0.25 and Na 50.

After 13 days of irrigation with the full-strength nutrient solution, plants of 31 days-old and eight true leaf pairs were subjected to NaCl stress or drought stress at Ψ_w_ of 0, -100, -250 and − 450 kPa. The experimental water potentials corresponded to 0, 25, 61 and 110 mM NaCl, respectively for salinity stress and to 100%, 23.6%, 14.1% and 10.2% field capacity, respectively for drought stress (equivalent to 20, 4.71, 2.83, 2.04 g water per 100 g sand, respectively). The water holding capacity of the sand used as growth medium was estimated as 20% (w/w). To achieve the experimental ψ_w_ of salinity and drought stresses, the formula of [22] and the ψ_w_-water content curves of [23], respectively were consulted. The experimental levels of stress were based on a previous study by the authors [15], which investigated the impact of a wide range of NaCl salinity stress and PEG water stress (down to -600 kPa) on seed germination and seedling growth of sesame. To ensure homogenous supply of water and nutrients, irrigation was dispended over four evenly-spaced daily intervals, with the same dose of nutrients throughout. This implied proportional increase in strength of the nutrient solution as the supply of water decreased in drought stress. The nutrient solution was prepared as a stock solution “Stock NS” so as 1 ml of which will provide the nutrient supply for one pot per day. The scheduled supply of nutrient solution, water (for drought stress) and NaCl (for salinity stress) is shown in Table 1.

**Table 1:** Supply of stock nutrient solution “Stock NS”, water and NaCl solution to the sand culture to obtain the experimental water potentials with the same dose of nutrients under the impact of salinity stress and drought stress

	kPa			
	0.00	-100	-250	-450
Salinity stress				
mM NaCl	0	25	61	110
Water (ml) before addition of stocks	200	200	200	200
Stock NS. (ml per pot per day)	1.0	1.0	1.0	1.0
2 M NaCl (ml per pot per day)	0	2.75	6.71	12.1
Final volume (ml)	220	220	220	220
Working solution (ml per pot per 6 h)	55	55	55	55
Drought stress				
Water content of sand (% DW)	20	4.71	2.83	2.04
Water content of sand (% field capacity)	100	23.6	14.1	10.2
Stock NS (ml per pot per day)	1.0	1.0	1.0	1.0
Water (ml per pot per day), up to	220	51.92	31.02	22.44
Working solution (ml per pot per 6 h)	55	12.98	7.76	5.61

Plants were grown in the greenhouse at the Faculty of Science, Damietta University during the period from July to August 2023. Irradiance ranged from 1500 to 2000 µmol m^− 2^ s^− 1^ from natural sunlight. Maximum air temperature ranged from 32 to 38 °C and minimum temperature from 22 to 25 °C in a 14/10 h light/dark period and relative humidity range of 70–80%. Plants were harvested six days after imposition of stress, when they were 37 days old and bearing about 12 leaf pairs under no stress. Because of vigorous plant growth during the treatment period, a stress period of six days was enough to express the genotypic differences as well as the differential responses to type of stress in terms of plant morphology and growth. During this six-day period, the control plants produced up to four additional leaves. Just before harvest, measurements of leaf angle, leaf dimensions, photosynthetic pigments and gas exchange were carried out in the third youngest leaf pair. Leaf orientation was measured as the angle between the stem and petiole by using a protractor. Plants were thoroughly extracted from sand, washed, separated into leaves, stem and root, blotted gently and fresh weights were recorded. An aliquot of the fresh leaf biomass was kept frozen at -80 °C for estimation of photosynthetic pigments and carbohydrate fractions. The fresh plant material was dried at 80 °C for 48 h and dry weights were corrected for the leaf portions kept frozen.

### Plant analysis

#### Estimation of photosynthetic pigments

Photosynthetic pigments were determined according to the method described by [24]. Frozen leaf tissue was homogenized in 80% acetone using cold mortar and pestle in dim light with traces of magnesium carbonate to neutralize the plant acids. The slurry was centrifuged and the clear extract brought up to volume with 80% acetone where the absorbance was read at 470, 646 and 663 nm using a UNICO 7200 series Spectrophotometer. The concentrations of chlorophyll a (Chl a), chlorophyll b (Chl b) and carotenoids (Carot) were calculated (µg ml^− 1^) according to the following equations of [24]:

$$\mathrm{Chl}\;\text a\;=\;12.21\;{\text E}_{663}-2.81\;{\text E}_{646}$$

$$\mathrm{Chl}\;\text b\;=\;20.13\;{\text E}_{646}-5.03\;{\text E}_{663}$$

$$\begin{aligned}\mathrm{Carot}\;&=\;\frac{\left(1000\;E_{470}\;-\;3.27\;Chla-104\;Chl\;b\right)}{229}\end{aligned}$$

Concentrations were presented as µmol pigment g^− 1^ leaf FW.

#### Estimation of gas exchange parameters

Photosynthesis rate (A), stomatal conductance to CO_2_ (G_s_), substomatal CO_2_ concentration (C_i_) and transpiration rate (E) of the third youngest leaf pair were measured at 10:00 a.m. using an LCA-4 portable gas exchange system (Analytical Development Company Ltd, England). Measurements were conducted–according to manufacturer’s instructions–with leaf area of 6.25 cm^2^, leaf chamber CO_2_ concentration of 390 ppm at chamber temperature of 44 °C and photosynthetic photon flux density of 1500 µmol m^− 2^ s^− 1^.

#### Estimation of carbohydrate fractions

Soluble sugars, starch, hemicellulose and pectic substances were sequentially assayed in the leaves. For extraction of soluble sugars (SS), a known weight of the frozen leaf material was grinded to a fine powder in liquid nitrogen, then thoroughly extracted in 1.2 ml of 80% ethanol and kept at -4 °C overnight [25]. The mixture was centrifuged at 8,000 *× g* for 10 min, and the supernatant was quantitatively transferred to clean Eppendorf tubes. The supernatant was then evaporated to dryness at 70 °C and re-dissolved in distilled water for determination of soluble sugars. An aliquot of the soluble sugar extract was completed to 1 ml with distilled water, then carefully mixed with 3 ml of the anthrone reagent and heated at 80 °C for 10 min in a water bath. The mixture was cooled for 30 min in an ice bath and absorbance was read at 623 nm against the reagent blank.

The plant debris left after ethanolic extraction was used for determination of the insoluble carbohydrate fractions. For estimation of starch, the debris remained after extraction of soluble sugars was re-suspended in 1.5 ml of 3% HCl with boiling for 3 h and the mixture was centrifuged at 8,000 *× g* for 10 min [26]. The liberated glucose units were estimated by the anthrone method as mentioned above. For estimation of hemicellulose, the residue left after extraction of starch was re-suspended in 1.2 ml of 5% H_2_SO_4_, boiled for 2.5 h and then centrifuged at 8,000 *× g* for 10 min. The supernatant was quantitatively transferred to clean Eppendorf tubes, and the liberated glucose units were estimated by the anthrone method [27]. For estimation of pectic substances, the residue left after extraction of hemicellulose was re-suspended in 1 ml of 72% H_2_SO_4_ at 4 °C for 24 h, then centrifuged at 8,000 *× g* for 10 min. The supernatant was quantitatively transferred to clean Eppendorf tubes, and the liberated glucose units were estimated by the anthrone method [27]. The anthrone reagent was prepared by dissolving 1.67 g anthrone in one liter of 80% H_2_SO_4_ (v/v) for 8.6 mM anthrone. The different carbohydrate fractions were estimated with reference to a glucose calibration curve in the range of 0–100 µg ml^− 1^ and expressed as glucose equivalents per leaf fresh weight (mg Gluc g^− 1^ leaf FW).

#### Definitions and calculations

The photosynthetic water use efficiency (WUE) was defined as the ratio of photosynthesis rate (A) to transpiration rate (E) [1]. RWR, StWR and LWR refer to the proportions of plant biomass allocated to root, stem and leaves respectively. Similarly, Chl a ratio, Chl b ratio and Carot ratio refer to the proportions of Chl a, Chl b and Carot, respectively in the total leaf pigment content. The threshold stress was defined as the highest level of stress (kPa) leading to non-significant reduction in plant growth and performance; rather, the threshold might encompass a beneficial effect.

### Experimental design and statistical analysis

The experiment was factorial with three replications in a completely randomized design. The main factors were: (1) sesame cultivar with two levels: Sohg and Shnd, (2) type of abiotic stress with two levels: salinity and drought and (3) severity of stress in terms of Ψ_w_ of the sand culture with four levels: 0, -100, -250 and − 450 kPa. Data were subjected to three-way ANOVA using SPSS version 22, to assess the effects of the main factors and their interactions on sesame growth and performance. Mean separation was performed according to the Duncan’s multiple range test at *P* < 0.05. The correlations between plant growth and performance were depicted in terms of Pearson correlation coefficient at *P* < 0.05, *P* < 0.01 and *P* < 0.001 for significant, highly significant and very highly significant correlations, respectively.

## Results

### Plant growth

**Fig. 1:**
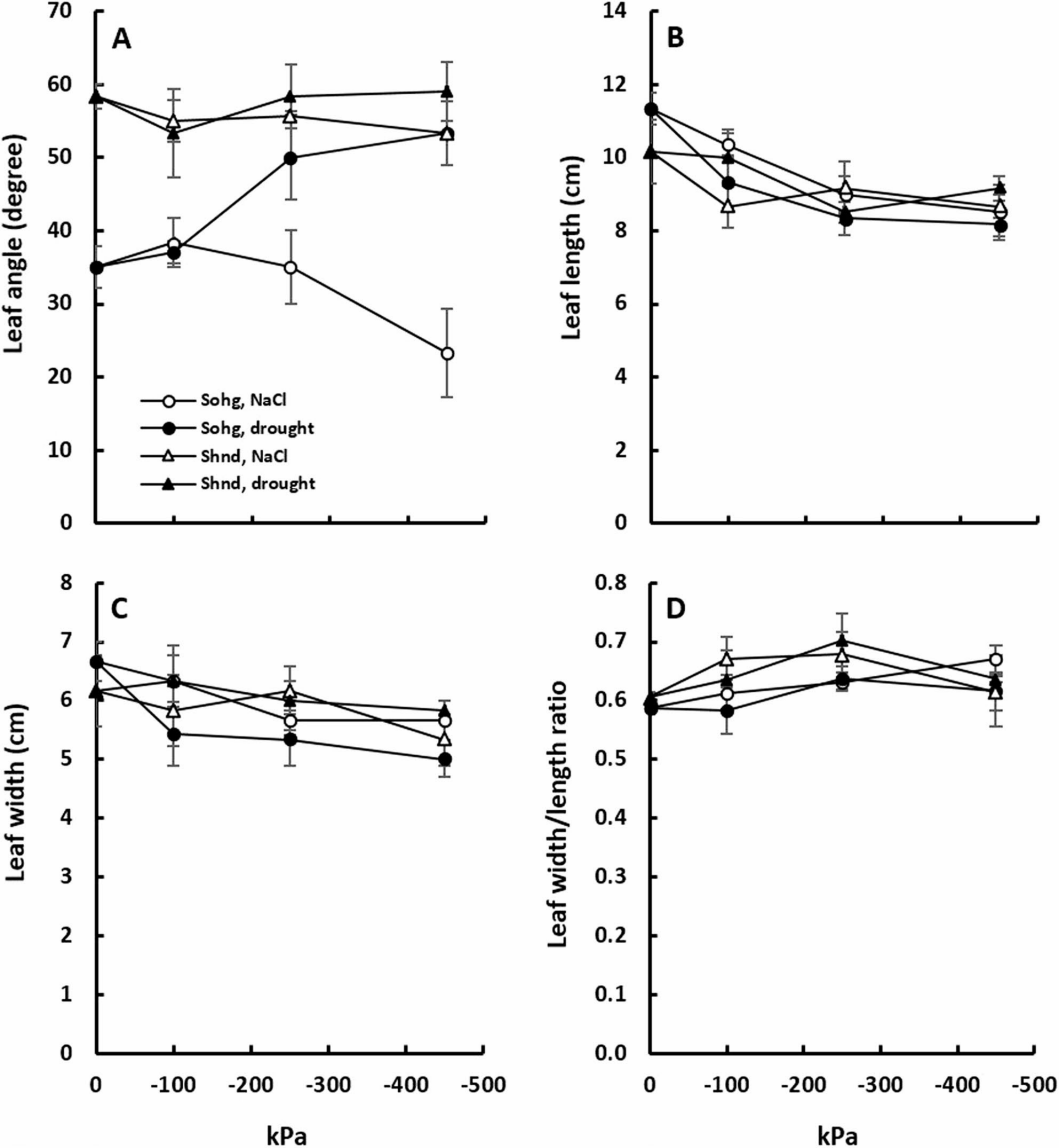
Effect of salt stress and drought stress on leaf angle (**A**), leaf length (**B**), leaf width (**C**) and leaf width/length ratio (**D**) of cvs Sohg and Shnd of sesame. Each value is the mean of three replicates ± SE

The most affected growth attributes, justified by magnitude of the F ratio, was leaf angle, while the least affected one was leaf dimensions (Tables 2 and 3). In absence of stress, Shnd exhibited horizontal leaf position relative to Sohg. The effect of stress on leaf orientation was observed only in Sohg, which experienced 39% reduction in leaf angle by salt stress versus 52% increase by drought stress across the whole range of ψ_w_; this in contrast to non-significant decrease and increase, respectively in Shnd (Fig. 1A). Both salinity stress and water stress led to progressive decreases in leaf length and width in Sohg versus non-significant reductions in Shnd (Fig. 1B, C). Consequently, stress led to a mild but progressive increase in the leaf width/length ratio of Sohg but to a transient top at moderate stress, followed by a decrease at severe stress in Shnd (Fig. 1D).

**Table 2:** Three-way ANOVA showing the effect of the main factors: sesame cultivar (Cv), type of stress (Stress) and water potential of the medium (kPa) and their interactions on leaf morphology and water content of plant organs

Source of variation	df	Leaf angle		Leaf length (L)		Leaf width (W)		Leaf W/L ratio		Root water		Shoot water	
		F	*P*	F	*P*	F	*P*	F	*P*	F	*P*	F	*P*
Cv	1	83.55	0.000	0.611	0.440	0.361	0.552	3.121	0.087	7.651	0.009	0.378	0.543
Stress	1	10.16	0.003	0.126	0.725	0.361	0.552	0.269	0.608	39.24	0.000	0.499	0.485
kPa	3	0.709	0.554	11.16	0.000	3.252	0.034	3.003	0.045	11.18	0.000	4.894	0.007
Cv × Stress	1	5.491	0.025	1.823	0.186	2.373	0.133	0.488	0.490	1.574	0.219	1.775	0.192
Cv × kPa	3	0.919	0.443	1.715	0.184	1.053	0.383	1.291	0.294	2.576	0.071	3.063	0.042
Stress × kPa	3	5.133	0.005	0.423	0.738	0.065	0.978	0.430	0.733	20.02	0.000	1.536	0.224
Cv × Stress × kPa	3	2.150	0.113	0.880	0.462	0.627	0.603	0.375	0.772	1.297	0.293	2.771	0.058

**Table 3:** Three-way ANOVA showing the effect of the main factors: sesame cultivar (Cv), type of stress (Stress) and water potential of the medium (kPa) and their interactions on biomass and biomass partitioning among plant organs

Source of variation	df	Root dry weight		Stem dry weight		Leaf dry weight		RWR		StWR		LWR	
		F	*P*	F	*P*	F	*P*	F	*P*	F	*P*	F	*P*
Cv	1	0.498	0.486	0.827	0.370	0.691	0.412	1.360	0.252	4.987	0.033	1.043	0.315
Stress	1	96.52	0.000	21.73	0.000	48.02	0.000	272.2	0.000	25.20	0.000	69.17	0.000
kPa	3	1.368	0.270	21.99	0.000	30.42	0.000	10.45	0.000	4.264	0.012	0.683	0.569
Cv × Stress	1	0.778	0.384	0.282	0.599	0.036	0.851	0.002	0.963	0.643	0.428	0.428	0.518
Cv × kPa	3	1.610	0.206	8.508	0.000	1.172	0.336	1.358	0.273	5.591	0.003	1.950	0.141
Stress × kPa	3	41.62	0.000	7.303	0.001	14.22	0.000	48.01	0.000	4.181	0.013	13.78	0.000
Cv × Stress × kPa	3	0.167	0.918	0.442	0.724	0.371	0.775	0.016	0.997	1.086	0.369	0.945	0.431

**Fig. 2:**
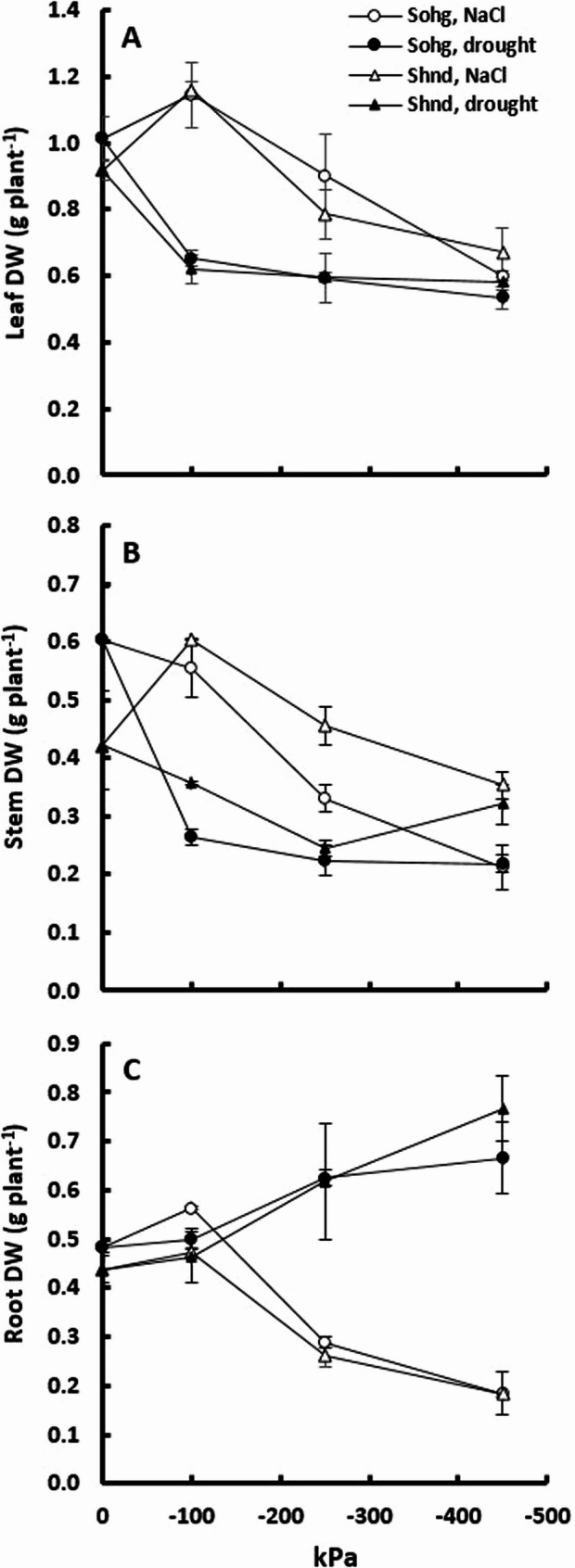
Effect of salt stress and drought stress on dry weight of leaves (**A**), stem (**B**) and root (**C**) of cvs Sohg and Shnd of sesame. Each value is the mean of three replicates ± SE

Whereas the dry weights of root and leaves were comparable in the two cultivars irrespective of the stress regime, stem dry weight was higher in Sohg than Shnd in absence of stress but the reverse was true under stress. The differential impact of salinity stress and drought stress was more evident–with diminished genotypic variability–on leaf and root biomass compared to stem biomass. Lowering ψ_w_ from 0 to -450 kPa reduced leaf dry weight of Sohg and Shnd by an average of 43%; however, the reduction occurred beyond a beneficial threshold salinity of -100 kPa but progressively under drought stress (Fig. 2A). By contrast, the differential impact of salinity and drought stresses was lacking on stem biomass, rather the genotypic difference was marked with overall stronger impact in Sohg than Shnd (Fig. 2B). Increasing severity of salinity stress beyond a threshold of -100 kPa down to -450 kPa reduced root dry weight of Sohg and Shnd by an average of 65%; meanwhile drought stress led to progressive increases of 38% and 75% in Sohg and Shnd, respectively (Fig. 2C).

Shoot water content was non-significantly affected neither by the genotype nor stress regime, except for the significant reduction only in Shnd under severe salinity. By contrast, the genotypic difference emerged for root water content, with overall higher values in Sohg than Shnd particularly under stress, and stronger reduction under drought than salinity in the two cultivars (Table 4).

**Table 4:** Effect of salt stress and drought stress on water content of shoot and root of cvs Sohg and Shnd of sesame. Each value is the mean of three replicates ± SE. Means with common letters are not significantly different at *p* ≤ 0.05

Stress and water potential (kPa)	Shoot water (% FW)	Root water (% FW)
	Sohg	
Salt stress		
0	86.02 ± 0.10^a^	91.17 ± 0.86^ab^
-100	87.22 ± 0.16^a^	91.81 ± 0.87^ab^
-250	86.37 ± 0.39^a^	92.03 ± 0.30^ab^
-450	86.63 ± 1.60^a^	94.02 ± 1.26^a^
Drought stress		
0	86.02 ± 0.10^a^	91.17 ± 0.86^ab^
-100	87.40 ± 0.49^a^	93.89 ± 0.09^a^
-250	86.02 ± 0.29^a^	89.65 ± 1.30^bc^
-450	85.49 ± 0.27^a^	84.79 ± 1.57^d^
	Shnd	
Salt stress		
0	86.69 ± 0.42^a^	91.86 ± 0.56^ab^
-100	86.84 ± 0.44^a^	92.40 ± 0.70^ab^
-250	88.17 ± 0.20^a^	91.08 ± 0.89^abc^
-450	80.50 ± 3.44^b^	91.35 ± 1.11^ab^
Drought stress		
0	86.69 ± 0.42^a^	91.86 ± 0.56^ab^
-100	87.43 ± 0.16^a^	89.74 ± 0.31^bc^
-250	86.51 ± 0.64^a^	88.02 ± 2.48^c^
-450	85.80 ± 1.08^a^	81.62 ± 0.84^e^

**Fig. 3:**
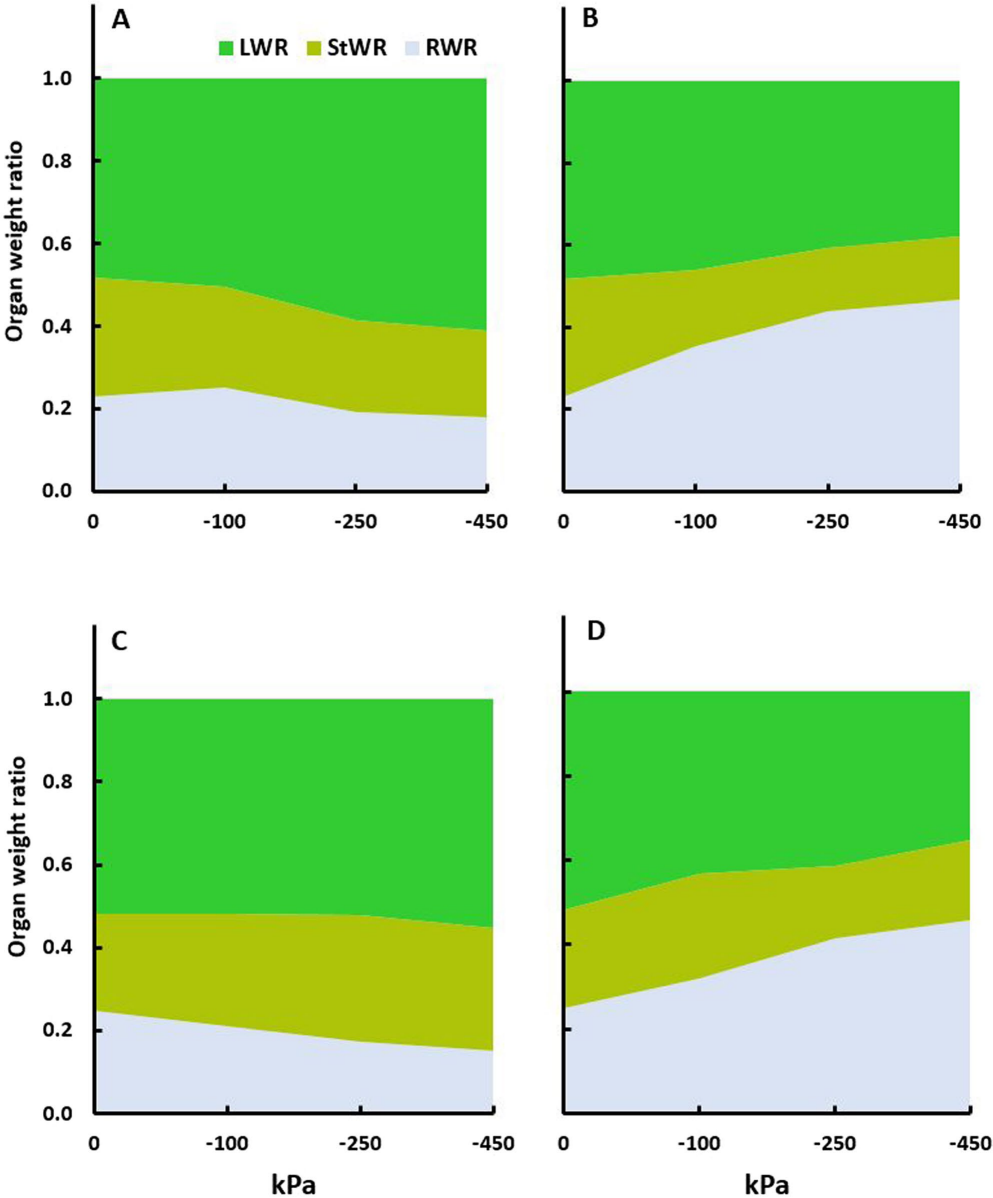
Effect of salt stress and drought stress on biomass partitioning among leaves, stem and root of sesame: (**A**) Sohg NaCl, (**B**) Sohg drought, (**C**) Shnd NaCl and (**D**) Shnd drought. Values are the means of three replicates. LWR, StWR and RWR are the proportions of the plant dry matter allocated to leaves, stem and root, respectively

The differential impact of salinity and drought stresses on dry weights of plant organs resulted in alteration of biomass partitioning among leaves, stem and root. Under salinity stress, Sohg increased allocation of biomass to leaves at the expense of stem and root; whereas Shnd increased biomass allocation to the stem at the expense of root with marginal increase in leaf proportion. By contrast, under drought stress both cultivars increased allocation of biomass to root at the expense of foliage (stem and leaves) (Fig. 3).

### Photosynthetic pigments

**Fig. 4:**
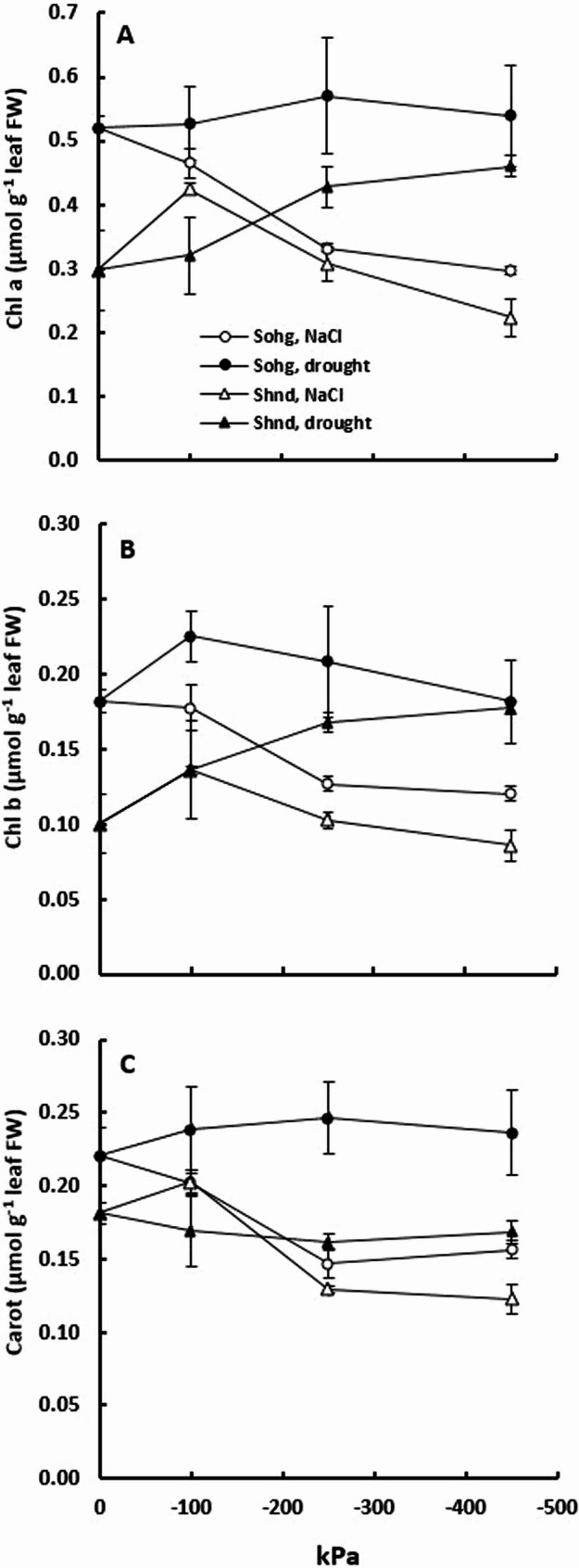
Effect of salt stress and drought stress on the content of Chl a (**A**), Chl b (**B**) and Carot (**C**) of cvs Sohg and Shnd of sesame leaves. Each value is the mean of three replicates ± SE

**Fig. 5:**
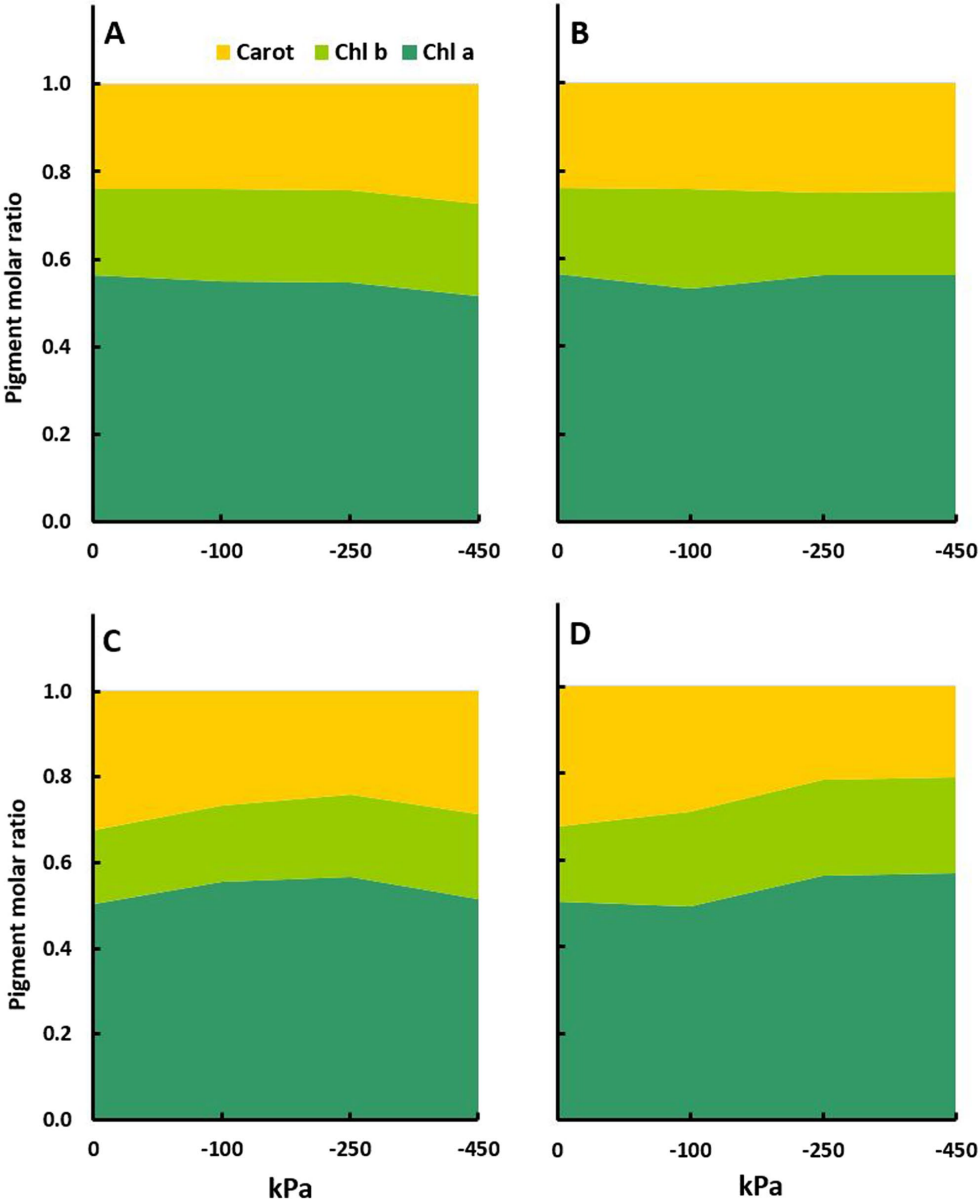
Effect of salt stress and drought stress on pigment partitioning among Chl a, Chl b and Carot of sesame leaves, in terms of the molar ratio of each pigment in the total pigment content: (**A**) Sohg NaCl, (**B**) Sohg drought, (**C**) Shnd NaCl and (**D**) Shnd drought. Values are the means of three replicates

The contents of Chl a, Chl b and Carot were significantly higher in Sohg than Shnd; the genotypic difference was either most evident under mild stress (chlorophylls) or maintained under all levels of stress (Carot) (Table 5). The impact of salinity on photosynthetic pigments was more aggressive than that of drought; in fact, the latter exerted a beneficial effect in Shnd. Salinity stress significantly reduced pigment content in the two cultivars; but whereas the reductions were progressive in Sohg, they occurred beyond a threshold of -100 kPa in Shnd. Meanwhile, drought stress non-significantly affected pigment content of Sohg but significantly increased chlorophyll content of Shnd without effect on Carot content (Fig. 4). Generally, partitioning of photosynthetic pigments among Chl a, Chl b and Carot showed mild response to treatments, except for: (1) salinized Sohg–where Carot proportion was increased at the expense of Chl a, and (2) drought-stressed Shnd–where chlorophyll proportions were increased at the expense of Carot (Fig. 5).

**Table 5:** Three-way ANOVA showing the effect of the main factors: sesame cultivar (Cv), type of stress (Stress) and water potential of the medium (kPa) and their interactions on photosynthetic pigments and pigment partitioning in sesame leaves

Source of variation	df	Chl a		Chl b		Carot		Chl a ratio		Chl b ratio		Carot ratio	
		F	*P*	F	*P*	F	*P*	F	*P*	F	*P*	F	*P*
Cv	1	26.05	0.000	15.66	0.000	29.73	0.000	2.662	0.113	1.854	0.183	5.011	0.032
Stress	1	16.14	0.000	12.02	0.002	16.45	0.000	0.109	0.744	2.567	0.119	1.335	0.256
kPa	3	0.807	0.499	1.077	0.373	5.003	0.006	1.682	0.190	3.960	0.017	3.096	0.041
Cv × Stress	1	2.142	0.153	0.104	0.750	7.180	0.012	0.487	0.490	6.970	0.013	0.569	0.456
Cv × kPa	3	1.835	0.161	1.332	0.281	0.281	0.839	2.580	0.071	2.882	0.051	5.485	0.004
Stress × kPa	3	6.889	0.001	2.257	0.101	5.215	0.005	3.792	0.020	1.553	0.220	1.793	0.168
Cv × Stress × kPa	3	0.699	0.560	0.422	0.738	1.059	0.380	0.383	0.766	1.096	0.365	0.686	0.567

### Gas exchange

**Fig. 6:**
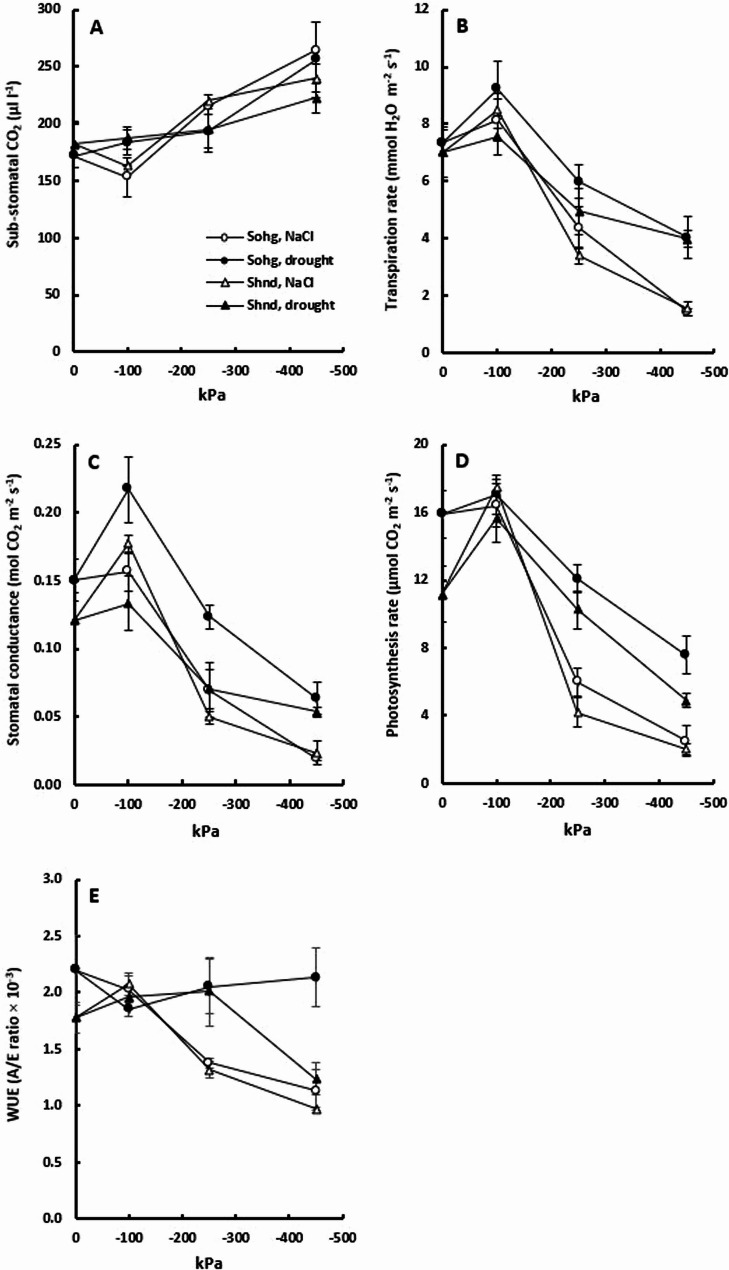
Effect of salt stress and drought stress on gas exchange parameters of leaves of cvs Sohg and Shnd of sesame: (**A**) substomatal CO_2_ concentration [C_i_], (**B**) transpiration rate [E], (**C**) stomatal conductance [G_s_], (**D**) photosynthesis rate [A], and (**E**) water use efficiency [WUE] (the A/E ratio). Each value is the mean of three replicates ± SE

Inspection of the F ratio and the associated P values reveals that the most affected gas exchange parameters were the rate of photosynthesis (A), followed by stomatal conductance (G_s_) and transpiration rate (E); while the least affected one was substomatal CO_2_ concentration (C_i_) (Table 6). In contrast to the increased C_i_ by stress, both of E, G_s_ and A were lowered. The genotype- stress interaction was evident on C_i_, with stronger impact of salinity than drought and in Sohg than Shnd. Under salinity stress, the increases in C_i_ across the whole range of ψ_w_ amounted to 54% and 39% in Sohg and Shnd, respectively beyond − 100 kPa threshold versus lower but progressive increases under drought stress (Fig. 6A). By contrast, both E, G_s_ and A showed marked differential response to type of stress with no genotypic difference. The reduction in E amounted to 77.5% under salinity stress and 47.5% under drought stress as an average for the two cultivars post a threshold of -100 kPa (Fig. 6B). The genotypic gap in G_s_ and A was marked, in favor of Sohg, in absence of stress but were narrowed under stress. Lowering ψ_w_ beyond a threshold of -100 kPa down to -450 kPa reduced G_s_ and A by 85% under salinity stress and by 57% under drought stress as averages for the two cultivars (Fig. 6C, D).

**Table 6:** Three-way ANOVA showing the effect of the main factors: sesame cultivar (Cv), type of stress (Stress) and water potential of the medium (kPa) and their interactions on the gas exchange parameters of sesame leaves

Source of variation	df	Substomatal CO_2_ (C_i_)		Transpiration rate (E)		Stomatal conductance (G_s_)		Photosynthesis rate (A)		WUE (A/E ratio)	
		F	*P*	F	*P*	F	*P*	F	*P*	F	*P*
Cv	1	0.071	0.791	2.307	0.139	11.19	0.002	13.54	0.001	6.306	0.017
Stress	1	0.089	0.768	10.68	0.003	7.701	0.009	17.55	0.000	10.10	0.003
kPa	3	17.43	0.000	61.65	0.000	61.56	0.000	96.24	0.000	10.02	0.000
Cv × Stress	1	0.077	0.783	0.749	0.393	6.509	0.016	1.068	0.309	0.824	0.371
Cv × kPa	3	1.247	0.309	0.504	0.682	1.016	0.398	2.866	0.052	2.452	0.081
Stress × kPa	3	1.836	0.161	3.583	0.024	1.688	0.189	8.000	0.000	5.336	0.004
Cv × Stress × kPa	3	0.016	0.997	0.567	0.641	2.361	0.090	0.353	0.787	1.070	0.376

Water use efficiency (WUE) was higher in Sohg than Shnd both in absence of stress and under severe drought stress but was comparable in the two cultivars under salinity stress and moderate drought stress. Salinity stress significantly reduced WUE in the two cultivars, either progressively in Sohg or beyond − 100 kPa threshold in Shnd. Meanwhile, the decrease in WUE was less severe under drought stress, and was either non-significant in Sohg or moderate beyond a threshold of -250 kPa in Shnd (Fig. 6E).

### Sugar fractions

**Fig. 7:**
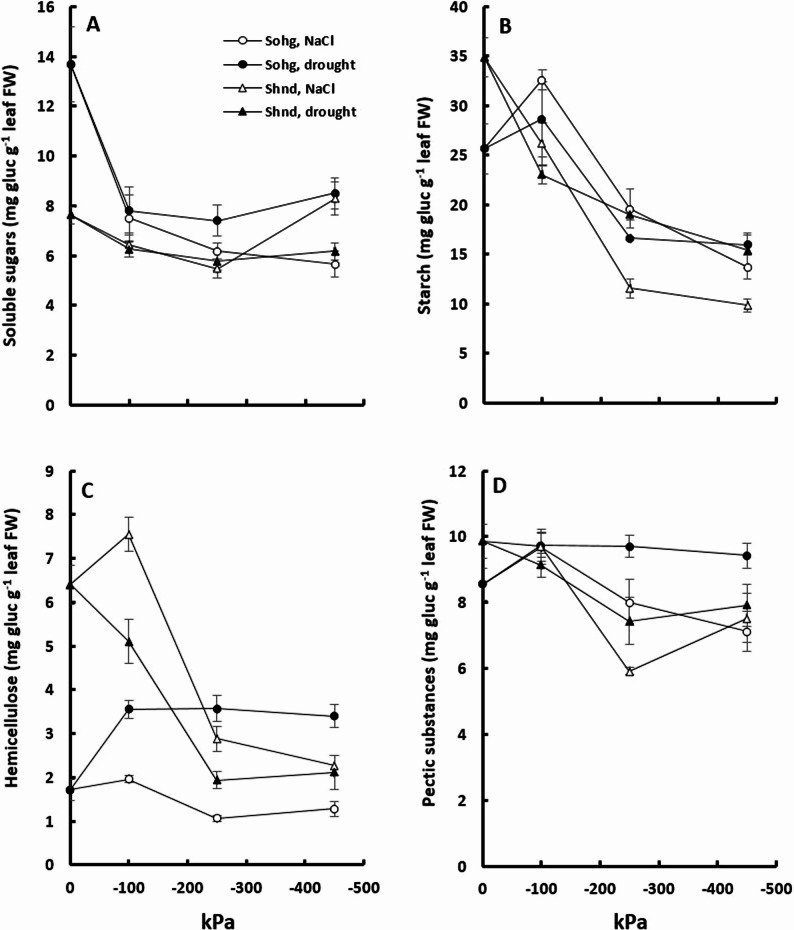
Effect of salt stress and drought stress on the carbohydrate fractions in the leaves of cvs Sohg and Shnd of sesame: (**A**) soluble sugars, (**B**) starch, (**C**) hemicellulose, and (**D**) pectic substances. Each value is the mean of three replicates ± SE

ANOVA revealed that among the assayed sugar fractions, hemicellulose was the most affected component, followed by soluble sugars, whereas pectic substances were the least affected one (Tables 7 and 8). Soluble sugar (SS) content of leaves was markedly higher in Sohg than Shnd both in absence of stress and under drought stress; but the stronger impact of salinity stress on Sohg than Shnd rendered SS content of salinized leaves higher in Shnd than Sohg. Whereas SS content of Sohg leaves experienced 58% and 38% reductions under salinity stress and drought stress, respectively, SS content of Shnd leaves experienced overall non-significant changes under stress, despite approaching a minimum at -250 kPa (Fig. 7A).

**Table 7:** Three-way ANOVA showing the effect of the main factors: sesame cultivar (Cv), type of stress (Stress) and osmotic potential of the medium (kPa) and their interactions on the carbohydrate fractions of sesame leaves

Source of variation	df	Soluble sugars		Starch		Hemicellulose		Pectic substances	
		F	*P*	F	*P*	F	*P*	F	*P*
Cv	1	31.48	0.000	0.243	0.625	181.9	0.000	2.811	0.103
Stress	1	0.642	0.429	0.513	0.479	4.795	0.036	6.783	0.014
kPa	3	27.92	0.000	79.90	0.000	58.64	0.000	11.74	0.000
Cv × Stress	1	4.540	0.041	4.050	0.053	64.88	0.000	1.715	0.200
Cv × kPa	3	13.18	0.000	13.63	0.000	64.70	0.000	7.530	0.001
Stress × kPa	3	0.216	0.885	3.138	0.039	4.750	0.008	3.307	0.032
Cv × Stress × kPa	3	2.298	0.096	1.709	0.185	8.668	0.000	0.699	0.559

**Table 8:** Three-way ANOVA showing the effect of the main factors: sesame cultivar (Cv), type of stress (Stress) and osmotic potential of the medium (kPa) and their interactions on the partitioning of the different carbohydrate fractions in sesame leaves

Source of variation	df	Soluble sugars ratio		Starch ratio		Hemicellulose ratio		Pectic substances ratio	
		F	*P*	F	*P*	F	*P*	F	*P*
Cv	1	31.18	0.000	0.995	0.326	165.1	0.000	0.202	0.656
Stress	1	2.344	0.136	0.016	0.902	1.868	0.181	0.136	0.715
kPa	3	41.88	0.000	31.97	0.000	10.81	0.000	28.13	0.000
Cv × Stress	1	16.97	0.000	36.93	0.000	81.70	0.000	0.747	0.394
Cv × kPa	3	47.87	0.000	10.61	0.000	28.92	0.000	0.942	0.432
Stress × kPa	3	3.677	0.022	1.468	0.242	0.968	0.420	0.465	0.709
Cv × Stress × kPa	3	6.934	0.001	7.246	0.001	12.54	0.000	0.552	0.651

In contrast to SS, the leaf content of polysaccharides (starch, hemicellulose and pectic substances) was higher in Shnd than Sohg in absence of stress; but under stress, the genotypic difference was either diminished or turned in favor of Sohg. In Sohg, the reduction in starch content of leaves were comparable under salinity and drought, and in both stresses, it occurred beyond a threshold of -100 kPa; but in Shnd, the reduction was progressive, with stronger magnitude under salinity than under drought (Fig. 7B). The low hemicellulose content of Sohg leaves was subjected to moderate changes under stress: either a reduction under salinity or an increase under drought. By contrast, the high hemicellulose content of Shnd leaves experienced 70% average reduction across the whole range of ψ_w_; either progressively under drought stress or post a transient increase at -100 kPa under salinity stress (Fig. 7C). The content of leaf pectic substances showed significant reductions under salinity stress in Sohg as well as under salinity and drought stresses in Shnd, but drought stress led to non-significant increase in Sohg (Fig. 7D).

**Fig. 8:**
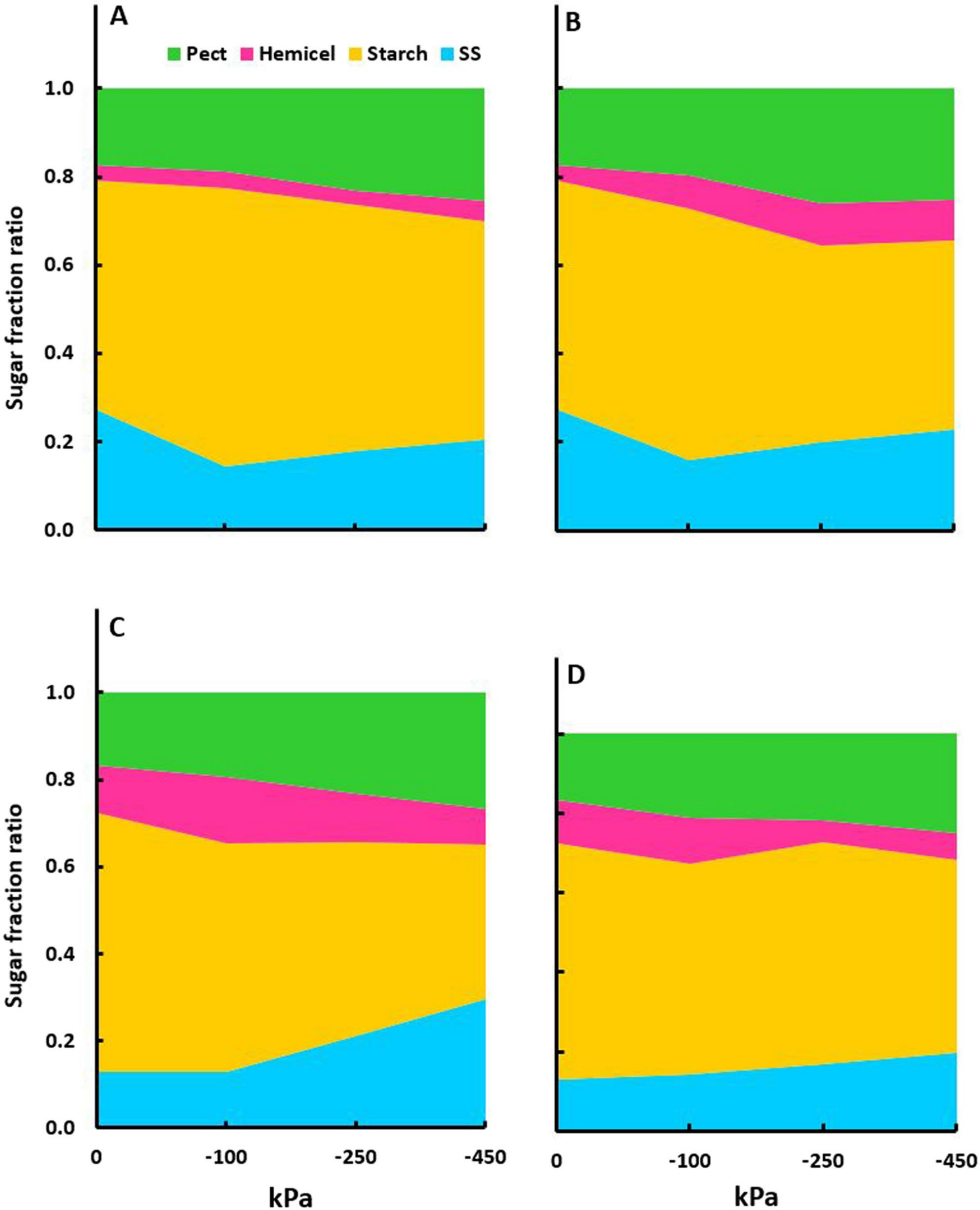
Effect of salt stress and drought stress on carbohydrate partitioning among soluble sugars, starch, hemicellulose and pectic substances in the leaves of Sohg NaCl (**A**), Sohg drought (**B**), Shnd NaCl (**C**) and Shnd drought (**D**) of sesame. Carbohydrate partitioning was assessed as the weight ratio of each component in the total carbohydrate content. Values are the means of three replicates

Starch constituted the greatest carbohydrate fraction of leaves (> 50%), followed by soluble sugars and pectic substances (about 20% each), with minor hemicellulose component. In Sohg, mild stress lowered the SS ratio in favor of starch, but severe stress led to the opposite effect; meanwhile, the fractions of pectic substances or those of pectic substances and hemicellulose were progressively increased with progression of stress (Fig. 8A, B). In Shnd, stress–particularly salinity–led to progressive increases in the ratios of SS and pectic substances at the expense of starch and hemicellulose (Fig. 8C, D).

## Discussion

Understanding the mechanisms the plant can adopt to withstand drought and salinity stresses is crucial for maintenance of crop productivity under unfavorable conditions. Stress effects on plant growth and performance are genotype-dependent but also depend on the severity, type and mode of imposition of stress. Because sesame is a moderately salt- and drought-tolerant crop [28, 29], it is often cultivated under the arid and semi-arid conditions of upper Egypt.

The present work reveals differential effect of salinity and drought stresses on shoot and root growth of the two sesame genotypes. Based on the current findings, it can be claimed that sesame foliage is more tolerant to salt stress than to drought stress, with overall better stress tolerance of cultivar Shnd than cultivar Sohg. The less severe impact of salinity stress on foliage biomass was manifested as the emergence of a threshold salinity that might encompass a beneficial effect, along with less-aggressive reduction under moderate salinity stress compared with drought stress. This finding can be viewed in light of the hypothesis that uptake and accumulation of salt ions, within limits, can aid in lowering ψ_w_ of plant tissues, thus allowing better water absorption from saline substrates than from arid substrates of the same water potential [30]. The beneficial effect of mild salinity to foliage growth and the lesser dehydrating effect of salinity than drought on root are lines of evidence supporting this hypothesis. However, the stronger dehydrating effect of salinity than drought on shoot suggests different patterns of osmotic susceptibility between shoot and root. In contrast to its aggressive effect on foliage biomass, drought stress improved root biomass of sesame. In apparent contradiction with our findings, decreased root volume and root hydraulic conductivity of wheat by PEG-drought stress and NaCl-salinity stress has been reported [31]. Thus, the response of root to drought stress imposed by artifacts such as PEG might differ from its response to physical drought imposed by water withholding. However, the reduction in root volume along with increased root density by drought reported by [32] implies either less severe impact of drought on root biomass than on root volume or even enhanced root biomass production by drought.

A functional equilibrium is always achieved by plants to balance their biomass allocation between root and shoot [1]. Accordingly, it is expected that plants invest more biomass in the root at the expense of foliage under water deficit to optimize acquisition of water and nutrients and to restrict transpirational water loss. The drought-induced reduction in plant biomass, reported for both wild and cultivated barley species, was attributed primarily to reductions in leaf biomass and area despite maintenance of meristematic activity inferred from the conserved number of leaves [33]. The present work suggests that while salinity favors allocation of plant biomass to the foliage at the expense of root, drought led to the opposite effect. However, this pattern was subjected to genotypic intervention. Whereas the favored biomass allocation to the shoot under salinity occurred in favor of either leaves (Sohg) or stem (Shnd), the favored biomass allocation to root under drought occurred at the expense of either stem and leaves (Sohg) or only leaves (Shnd). Drought has been claimed to increase root to shoot biomass ratio of plants [34]; specifically speaking, to favor allocation of plant biomass to root and stem at the expense of leaves [35]. By contrast, it has been reported that the more salinity robustness of root than shoot can lead to increased root/shoot ratio of salinized plants [36, 37]. Hence, it seems that the effect of stress on biomass allocation is controversial and depends on the experimental conditions and genotype [38]. As an example of the genotype intervention in modifying the effect of stress in biomass partitioning is the fact that leaf weight ratio was decreased in *Medicago sativa* [39] but increased in *Setaria viridis* [40] by drought.

**Fig. 9:**
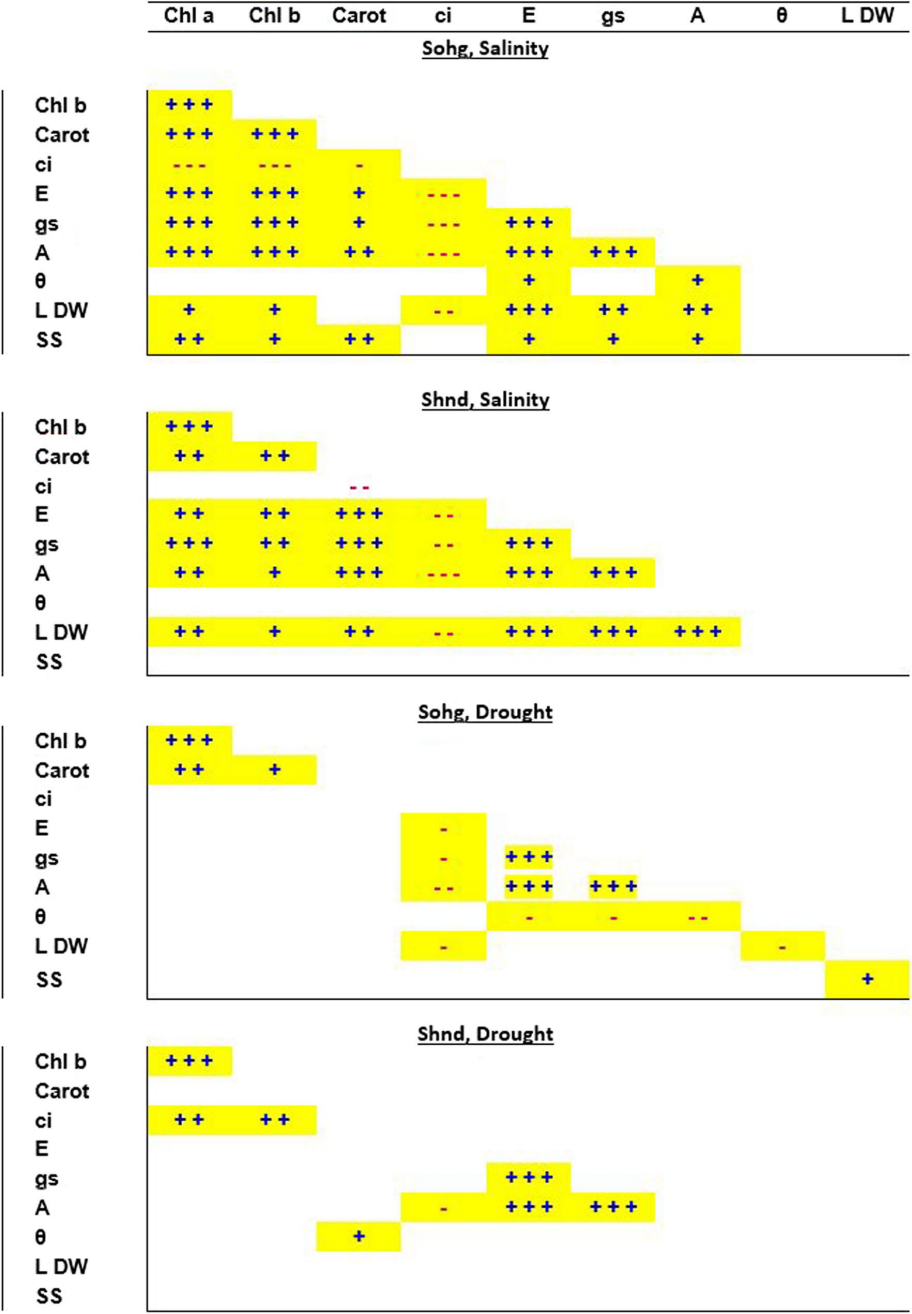
Correlation analysis showing the relationships between gas exchange parameters [photosynthesis rate (A), stomatal conductance (G_s_), transpiration rate (E) and substomatal CO_2_ concentration (C_i_)], leaf angle (θ), leaf pigments (Chl a, Chl b and Carot) and soluble sugars (SS) with leaf biomass (L DW) of cvs Sohg and Shnd of sesame under salinity stress and drought stress. **+ + +** and **- - -** denote very highly significant (*P* < 0.001) positive and negative correlation, respectively; **+ +** and **- -** highly significant (*P* < 0.01) correlation, **+** and - just significant (*P* < 0.05) correlation

The inherent horizontal leaf orientation of Shnd was marginally affected by stress. By contrast, the vertical leaf orientation of Sohg was differentially affected by salinity and drought stresses; becoming more vertical under salinity but less vertical under drought. These findings imply that, only in the stress-sensitive Sohg, the stress-induced alteration in leaf orientation, that is the vertical position under salinity and the horizontal position under drought, can be considered as signs of stress, mostly related to lowering in photosynthesis and transpiration rates (Fig. 9). Generally, whereas the biomass of vertical Sohg leaves were conserved under the impact of salinity stress, Shnd conserved stem biomass at the expense of the horizontal leaves (Fig. 3). Leaf orientation is an important factor in regulation of plant carbon–water–energy nexus to optimize photosynthesis and light interception under the impact of abiotic stress. Unfavorable environmental conditions can evoke leaf movements, such as reversible epinastic responses [41], which can aid in plant adaption to stress conditions via protecting the leaves from photoinhibition by reducing the leaf area exposed to solar irradiation [42]. It has been accepted that, in general, leaves become vertical in hot dry environments but more horizontal in mesic and light-limited environments [43].

The genotype-stress interaction on photosynthetic pigments of sesame leaves was manifested as a marked negative impact of salinity (being more severe in Sohg than Shnd), versus almost no effect in Sohg or even improvement in Shnd by drought. The symptoms of salt stress on plants include chlorosis and scorching of leaves [44]. However, the effect of salinity on chlorophyll content of wheat leaves seems to be genotype-dependent, being promotive in the salt-tolerant genotypes but inhibitory in the salt-sensitive ones [45]. Chlorophyll content of leaves has been reported to decrease under both salinity and drought stresses in *Robinia pseudoacacia* [46] and under drought stress in wheat [47], canola [48] and barley [49]. The increased photosynthetic pigments in the leaves of *Cynanchum acutum* under drought, in agreement with our findings, was linked to drought-induced phosphorus deprivation [35]. It has been proposed that leaf greening induced by phosphorus deficiency does not imply enhanced chlorophyll content but can be attributed to more severe reduction in leaf growth than chlorophyll formation. The observed reductions in leaf dimensions and chlorophyll content of sesame leaves under salinity stress points to a specific toxicity of salt ions in reducing chlorophyll content, probably via acceleration of chlorophyll degradation or retardation of its synthesis. By contrast, the trivial reduction in leaf dimensions along with the marked increase in chlorophyll content of leaves under drought stress points to enhanced chlorophyll content via acceleration of chlorophyll synthesis or diminished degradation. Furthermore, the present work suggests that abiotic stress can minimize the genotypic gap in chlorophyll content between the two sesame cultivars that is approximating the less green Shnd to the greener Sohg. The effect of stress on partitioning of photosynthetic pigments of sesame leaves among Chl a, Chl b and Carot was particularly evident in Shnd, in which stress can increase Chl a proportion at the expense of Carot. In accordance with the present findings, Chl a and Chl b contents were subjected to differential impact of salinity in rice leaves [50] and of drought in wheat leaves [47].

Photosynthesis rate (A), transpiration rate (E) and stomatal conductance (G_s_) are usually positively correlated to water potential of the medium [51]. Hindered photosynthesis under stress is usually linked to stomatal closure but can also result from non-stomatal (biochemical and photochemical) constraints [52]. The apparently matched patterns of stress-induced retardation in A, E and G_s_ of sesame leaves indicate that the reduced photosynthetic activity can be primarily attributed to the stress-induced stomatal closure and lowering of stomatal conductance (Fig. 9). However, thorough inspection of the gas exchange patterns reveals a probable role of non-stomatal limitations in controlling the photosynthetic efficiency of sesame leaves. The native photosynthetic efficiency (A) of Sohg was distinctly higher than that of Shnd; meanwhile, the genotypic difference in gas exchange capacity (E and G_s_) was relatively narrow. Furthermore, although A generally exhibited greater stress-susceptibility relative to E and G_s_, a fine genotype-stress interaction on A, E and G_s_ was observed. It can be claimed that A is more salinity-robust in Shnd but more drought-robust in Sohg, in contrast to E and G_s_ which showed the opposite trend. These facts point to fine tuning by non-stomatal limitations–that is the photochemical and biochemical processes–in controlling photosynthetic efficiency. Nevertheless, the stronger impact of severe salinity relative to severe drought on gas exchange of the two sesame cultivars refers to a specific effect of salt ions in interference with the photosynthetic machinery beside the osmotic effect shared by the two stresses. Furthermore, the stronger impact of salinity than drought on leaf gas exchange, in contradiction to the response of foliage biomass, refers to differential effect of salt ions on instantaneous gas exchange and foliage growth. Correlation analysis (Fig. 9) also revealed that A, E and G_s_ are positively correlated with each other but negatively correlated with C_i_. This trend along with the coincidence of minimal C_i_ and maximal A and G_s_ at mild salinity (Fig. 6) implies that: (1) stress can inhibit photosynthetic efficiency without effect on or even enhancement of respiration, and (2) mild salinity can enhance photosynthetic efficiency through increasing stomatal conductance. In agreement with our claim, the drought-induced stomatal closure with severe inhibition in photosynthesis occurred concomitant with mild inhibition or even promotion in respiration [35, 53].

Stomatal movement can tune photosynthesis and transpiration rates under stress in a way to increase water use efficiency (WUE). The present findings suggest that cv. Sohg–of high native photosynthesis potential–exhibited marked drought robustness. By contrast, cv. Shnd–of low photosynthesis potential–although benefited from mild stress experienced more susceptibility to severe drought. Both E and G_s_ benefited from mild salinity (in Shnd) and mild drought (in Sohg) but both were more prone to salinity stress than drought stress, with overall less stress-susceptibility compared with A. Therefore, abiotic stress seems to reduce WUE (the A/E ratio) of the two sesame cultivars, with stronger impact of salinity on Sohg and of drought on Shnd. Water-use efficiency has been reported to increase in *Robinia pseudoacacia* under salinity and drought stresses [46] and in *Vicia faba* under salinity stress [37]. The observed lowering in WUE of sesame under severe drought can be attributed either to a greater sensitivity of the photosynthetic machinery relative to transpiration in response to water deficit. Alternatively, progression of water loss in spite of stomatal closure is probable via cuticular transpiration as proposed by [54]. Although stomatal closure is a mechanistic adaptation to water deficit in a way to avoid desiccation arising from transpirational water loss [55], the closed stomata will certainly inhibit photosynthesis by blocking the influx of CO_2_ to the mesophyll with the probability of continuation of water loss via alternative avenues such as cuticular transpiration.

Abiotic stress can affect both cell wall composition and production of compatible solutes [56]. The inhibited photosynthesis under abiotic stress, concomitant with the unaffected or favored respiration, results in lowering of carbohydrate reserves of sesame leaves. The decrease in carbohydrate content can be assigned to the decline in the major carbohydrate fraction (starch), followed by soluble sugars with marginal changes in pectic substances. However, the two sesame cultivars adopted different patterns of carbohydrate fractionation in response to stress. In Sohg, abiotic stress induced reductions in the proportions of starch and soluble sugars in favor of either pectic substances alone (salinity) or pectic substances and hemicellulose (drought). By contrast, in Shnd the reduction occurred in starch and hemicellulose proportions in favor of soluble sugars and pectic substances. Thus, the shared stress-induced response, irrespective of the genotype and type of stress, is the reduced proportion of starch in favor of pectic substances. The increased proportions of soluble sugars and pectic substances in Shnd under the impact of drought stress can aid in drought tolerance by contributing either to osmotic adjustment (soluble sugars) or by increasing cell wall elasticity (pectic substances). Osmotic and elastic adjustment are alternative strategies for a species to acclimate to water stress. Cells with highly elastic walls contain more water at full turgor; hence, their volume can decrease more before the turgor-loss point is reached. Elasticity of the cell wall depends on the chemical interactions between cell wall components [1]. It has been proposed that pectin, the major polyanionic polysaccharide in the walls of growing cells, plays a critical role in cell wall expansion [57]. However, the increase in soluble sugar proportion for osmotic adjustment under stress is evident only in Shnd, despite the distinct severe reduction in total carbohydrate content. The relative contribution of either osmotic adjustment or cell wall elasticity might vary according to the severity of drought. For example, the role of osmotic adjustment, achieved primarily via accumulation of nitrate and sugars, in drought resistance of *Spartina alterniflora*, was evident under mild drought but the role of cel wall elasticity was prominent under severe drought [58].

## Conclusions

The shift in sesame biomass in favor of foliage at the expense of root under salinity stress is accompanied with low gas exchange efficiency while the preferential allocation of biomass to the root under drought produces highly efficient although small foliage. Only in the stress-sensitive cultivar, leaf orientation exhibited plasticity; where horizontal leaf orientation under salinity but vertical orientation under drought seems appropriate for efficient gas exchange. Abiotic stress can narrow the genotypic gap in chlorophyll content between the two seme cultivars. The reduced photosynthetic activity by severe stress versus enhanced activity under mild stress are in harmony with the response of stomatal conductance. However, the role of nonstomatal limitations emerges from the fine disagreement between the response patterns of photosynthesis and stomatal conductance to the genotype-stress interaction. The lowered WUE under stress can be attributed either to greater sensitivity of the photosynthetic machinery than transpiration or to progression of water loss despite of stomatal closure, probably via cuticular transpiration. The stress-induced lowering in leaf carbohydrate reserves can be primarily assigned to the decline in starch content; meanwhile, the increased proportions of soluble sugars and pectic substances can aid in drought tolerance by contributing either to osmotic adjustment (soluble sugars) or increased cell wall elasticity (pectic substances). This study delineated the physiological responses of two sesame genotypes to iso-osmotically-induced salinity and drought stresses, via employing a consistent irrigation regimen instead of abrupt water deprivation. Salinity and drought differentially modulated orientation and performance of leaf, with altered partitioning of photosynthates among functional and structural carbohydrates in a genotype-dependent manner. The outcomes of this hydroponic study deserve further evaluation of the impact of salinity and drought stresses employing a wider collection of sesame genotypes under field conditions for reliable practical recommendations. 

## Data Availability

All data of this study are cited within the paper; raw data and calculations will be available by the corresponding author on request. The details of the corresponding author are: Taha Mohamed El-Katony, Department of Botany and Microbiology, Faculty of Science, Damietta University, New Damietta City, Egypt. e-mail: [tmsoliman2000@yahoo.co.uk] , [tmsoliman@du.edu.eg].
